# Regulation of prepubertal dynorphin secretion in the medial basal hypothalamus of the female rat

**DOI:** 10.1111/jne.12810

**Published:** 2019-12-02

**Authors:** William L. Dees, Jill K. Hiney, Vinod K. Srivastava

**Affiliations:** ^1^ Department of Veterinary Integrative Biosciences College of Veterinary Medicine Texas A&M University College Station TX USA

**Keywords:** dynorphin, insulin‐like growth factor‐1, neurokinin B, puberty

## Abstract

The onset of puberty is the result of an increase in secretion of hypothalamic gonadotrophin‐releasing hormone (GnRH). This action is a result of not only the development of stimulatory inputs to its release, but also the gradual decrease in inhibitory inputs that restrain release of the peptide prior to pubertal onset. Dynorphin (DYN) is one of the inhibitory inputs produced in the medial basal hypothalamus (MBH); however, little is known about what substance(s) control its prepubertal synthesis and release. Because neurokinin B (NKB) increases in the hypothalamus as puberty approaches, we considered it a candidate for such a role. An initial study investigated the acute effects of an NKB agonist, senktide, on the secretion of DYN from MBH tissues incubated in vitro. In other experiments, central injections of senktide were administered to animals for 4 days then MBHs were collected for assessment of DYN synthesis or for the in vitro secretion of both DYN and GnRH. Because insulin‐like growth factor (IGF)‐1 has been shown to play an important role at puberty, additional animals received central injections of this peptide for 4 days to assess NKB and DYN synthesis or the in vitro secretion of NKB. The results obtained show that senktide administration up‐regulates the NKB receptor protein, at the same time as suppressing the DYN and its receptor. Senktide consistently suppressed DYN and elevated GnRH secretion in the same tissue incubates from both the acute and chronic studies. IGF‐1 administration caused an increase in NKB protein, at the same time as decreasing DYN protein. Furthermore, the central administration of IGF‐1 caused an increase in NKB release, an action blocked by the IGF‐1 receptor blocker, JB‐1. These results indicate that the IGF‐1/NKB pathway contributes to suppressing the DYN inhibitory tone on prepubertal GnRH secretion and thus facilitates the puberty‐related increase in the release of GnRH to accelerate the onset of puberty.

## 1 INTRODUCTION

The onset of puberty is the result of a complex series of events within the hypothalamus that culminates in the increased secretion of gonadotrophin‐releasing hormone (GnRH). This increase in GnRH release is the result of a gradual decline of the prepubertal inhibition restraining its secretion, as well as the enhanced development of excitatory components that subsequently stimulate the critical increase in the release of the peptide needed to attain maturity. Although much progress has been made over the years with respect to identifying the neural mechanisms responsible for the pubertal stimulation in GnRH secretion, less is known about what neural mechanism(s) contribute to the prepubertal restraint in GnRH release, and what might contribute to the removal of this inhibition during pubertal onset. Several neural substrates such as gamma amino butyric acid,^1^ neuroestradiol^2^ and β‐endorphin (β‐ENDO)^3^ are produced by neurones in the arcuate nucleus (ARC) of the medial basal hypothalamus (MBH) and are capable of inhibiting prepubertal GnRH release. Additionally, dynorphin (DYN), another peptide produced in the ARC, has also been shown to inhibit prepubertal GnRH and thus, luteinising hormone (LH) secretion.^4^, ^5^, ^6^ Other investigators have shown that the administration of anti‐DYN caused increased serum LH,^7^ and that a kappa opioid receptor (KOR‐1) antagonist increased the pulsatile secretion of LH and advanced puberty.^8^, ^9^ Conversely, we revealed that prepubertal alcohol administration stimulates the hypothalamic synthesis and secretion of DYN^10^, ^11^; hence, DYN is suggested to be an important component of the prepubertal inhibition in GnRH/LH release that accompanies the delayed pubertal development in rats,^12^, ^13^, ^14^ monkeys^15^, ^16^ and humans^17^, ^18^ caused by chronic alcohol use.

Although the above examples demonstrate that DYN is an inhibitor of prepubertal GnRH, additional information is required to determine what upstream substance(s) are involved in controlling its synthesis and release. We considered neurokinin B (NKB) a likely candidate for such a role because it is synthesised by neurones within the ARC,^19^, ^20^, ^21^ it is known to increase in the hypothalamus as puberty approaches^22^ and it contributes to the rise in prepubertal GnRH/LH secretion.^23^, ^24^ Therefore, the present study utilised both in vivo and in vitro approaches to first investigate whether the prepubertal administration of senktide, an NKB agonist, could concomitantly suppress DYN and elevate GnRH secretion and, if so, to determine the specificity and reversibility of the actions. Finally, because of the well‐documented role of insulin‐like growth factor (IGF)‐1 to stimulate prepubertal GnRH release,^25^ and drive the pubertal process,^26^, ^27^, ^28^ we assessed whether this peptide is capable of upstream actions to facilitate NKB synthesis and release.

## 2 MATERIALS AND METHODS

### 2.1 Animals for study

Adult female rats of the Sprague‐Dawley line were purchased from Charles River, bred and allowed to deliver pups normally in the Texas A&M University lab animal facility (College Station, TX, USA). Female pups were weaned at 21 days of age and housed four per cage (Nexgen individually ventilated caging system; Allentown Inc., Upper Freehold Township, NJ, USA) under a 12:12 hour light/dark cycle (lights on 6.00 am) at 23°C with access to food (Teklad Global 16% Protein rodent diet; Envigo, Huntingdon, UK) and water available ad libitum. All procedures performed on the animals were approved by the University Animal Care and Use Committee and in accordance with the NAS‐NRC Guidelines for the Care and Use of Laboratory Animals. Power analysis, along with extensive previous experience utilising these same protocols, determined the approximate number of animals needed for each experiment. Surgical anaesthesia was induced by an i.p. injection of 2.5% Tribromoethanol (0.5 mL/60 g body weight; catalogue no. T48402; Sigma‐Aldrich, St Louis, MO, USA). A stainless steel third ventricular (3V) cannula was stereotaxically implanted by moving 1.5 mm caudally from bregma and lowered 8.2 mm into the ventricle as described previously.^29^ On the day of surgery, cannula placement was verified by the presence of cerebral spinal fluid. At the end of the in vivo experiments, the cannulae placement was verified microscopically and animals with misplaced cannulae were not included in the experiments.

### 2.2 Effect of senktide on DYN release from the MBH

To assess DYN secretion, 29‐day‐old female rats were decapitated in the late juvenile phase of development and the MBH tissue block from each animal was dissected under a stereomicroscope, as described previously.^29^ Briefly, this tissue block extended from the caudal border of the optic chiasm to the mammillary bodies, caudally. This block was formed by making cuts along the borders of the hypothalamic sulci laterally, and along the border of the thalamus, dorsally. Each tissue block was incubated in vitro using a well‐established procedure.^25^, ^30^ Briefly, tissue vials were incubated in Locke's medium (2 mmol L^‐1^ Hepes, 154 mmol L^‐1^ NaCl, 5.6 mmol L^‐1^ KCl, 1 mmol L^‐1^ MgCl_2_, 6 mmol L^‐1^ NaHCO_3_, 10 mmol L^‐1^ glucose, 1.25 mmol L^‐1^ CaCl and 1 mg mL^‐1^ bovine serum albumin, pH 7.4) inside a Dubnoff shaker (95 cycles min^‐1^) at 37°C in an atmosphere of 95% O_2_ and 5% CO_2_. After an equilibration period of 30 minutes, the medium was discarded, and replaced with fresh medium for 30 minutes to establish basal release. Subsequently, these media were saved in microcentrifuge tubes then replaced with medium containing senktide (catalogue no. SML0764; Sigma‐Aldrich), an NKB agonist that binds to the neurokinin 3 receptor (NK3R). Thus, each vial represented a 30‐minute basal secretion control period, followed by a 30‐minute senktide response period. Senktide doses ranging from 0.1 to 1.0 µmol L^‐1^ were used and each repetition of the experiment contained incubation vials from all of the doses tested. After the final incubation, MBH fragments weighed a mean ± SEM of 18.5 ± 3.5 mg. The media samples were then collected and frozen for subsequent hormonal assessments. DYN and GnRH were measured from the same basal and treatment samples of each dose and the number of animals used was 16, 35, 32 and 35 for the 0, 0.1, 0.5 and 1 µmol L^‐1^ doses, respectively. The samples were then divided in half so that the remaining media in each sample was measured for either Kp (Kisspeptin) or ß‐ENDO using the respective enzyme‐linked immunoassays (ELISA) kit purchased from MyBiosource Inc. (San Diego, CA, USA).

### 2.3 Specificity of the effect of senktide on DYN release

The effect of senktide on the inhibition of DYN release was tested using the central administration of the NK3R antagonist, SB222200, which has been shown to block the NKB‐dependent activation of Kp neurones in the mouse^31^ and can delay female puberty.^32^ In this experiment, 25‐day‐old female rats were implanted with 3V cannulae as described previously.^29^ After 4 days of recovery, the animals were divided into the control group injected with vehicle (sterile Locke's medium) and the other half injected with SB222200^33^ (25 ng/4 µL of sterile Locke's medium; Bio‐Techne Brand, catalogue number: 1393/10; Tocris Bioscience, St Louis, MO, USA) into the 3V. Two hours post injection, the animals were decapitated and the 3V placement verified microscopically. The senktide dose utilised has been shown previously to stimulate LH release^34^ and advance puberty^35^; thus, the animals used were confirmed to be in the late juvenile stage of pubertal development as assessed by criteria described previously.^14^, ^36^ The MBH tissue blocks were dissected out as described above. Each MBH tissue block was incubated as above in medium only for 30 minutes to establish basal release. The medium was removed and stored then replaced with medium containing 1 µmol L^‐1^ senktide for another 30 minutes of incubation. At the end of this incubation, MBH fragments weighed a mean ± SEM of 15.2 ± 3.3 mg and the media samples were collected and saved for subsequent assessment of DYN and GnRH as described above. The total number of animals used was 25.

### 2.4 Effects of centrally administered senktide on protein synthesis within the MBH

In this experiment, 22‐day‐old female rats were implanted with 3V cannulae as described above. After 4 days of recovery, half of these 26‐day‐old rats were injected with senktide (Sigma‐Aldrich; 600 pmol/4 µL of saline) and the other half received an equal volume of sterile saline into the 3V daily for 4 days. All animals were killed 2 hours after their final injection, proper placement of the 3V cannula was microscopically verified and the animals confirmed to be in the late juvenile stage of pubertal development. The MBH tissues were dissected out as described above and the tissue samples were frozen on dry ice for protein assessments of NK3R, DYN, KOR‐1, β‐ENDO and Kp within the MBH by western blot analysis. The total number of animals used was 28.

### 2.5 Effects of centrally administered senktide on peptide release within the MBH

This experiment was conducted as above, except that peptide release was measured following an in vitro incubation of the MBH tissue fragment. After the 4 days of daily 3V injections of senktide or saline, the animals were killed 2 hours after the final injection and each MBH tissue block was dissected out and incubated in Locke's media as described above. After 30 minutes of incubation, MBH fragments weighed a mean ± SEM of 16.1 ± 4.6 mg. The media samples were collected and saved for subsequent assessment of DYN, β‐ENDO, Kp and GnRH released into the media. The total number of animals used was 26.

### 2.6 Effects of IGF‐1 on NKB in the MBH

In this experiment, 22‐day‐old female rats were implanted with 3V cannulae as described above. After 4 days of recovery, these 16‐day‐old rats were administered daily either sterile saline, or IGF‐1 (20 ng/3 µL of saline; catalogue number: cyt‐289‐c; Prospec Protein Specialists, Rehovot, Israel) for the next 4 days. The animals were killed by decapitation, 2 hours after the final injection of IGF‐1 or saline and proper placement of the cannula was verified. This dose of IGF‐1 has been shown previously to advance the timing of puberty in female rats^26^; therefore, only the animals confirmed to be in the late juvenile stage of development were used for subsequent assessment of NKB, DYN, ß‐ENDO and Kp protein by western blot analysis. The total number of animals used was 57.

**Figure 1:**
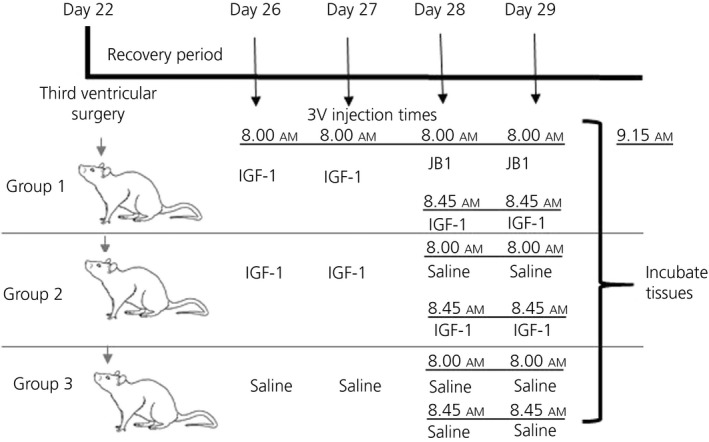
Dosing protocol and timeline to determine the effect of insulin‐like growth factor (IGF‐1 and its receptor antagonist (JB‐1) on neurokinin B release

Another experiment was conducted to assess NKB release in the presence or absence of the IGF‐1 receptor antagonist, JB‐1 (catalogue number: J3705; Sigma‐Aldrich). Here, 22‐day‐old female rats were implanted with 3V cannulae as described above. After 4 days of recovery, these 26‐day‐old rats were divided into three groups. Figure 1 illustrates the experimental protocol. Animals in Groups 1 and 2 received a 3V injection of IGF‐1 (20 ng/3 µL of saline) for two consecutive days. The animals in Group 3 received 3V injections of sterile saline (3 µL) for 2 days. On the third day of injections, at 8.00 am, animals in Group 1 received a 3V injection of JB‐1 (4 µg/4 µL of saline), a specific IGF‐1 receptor antagonist that blocks IGF‐1 phosphorylation of Akt^29^ and has been shown to block the steroid‐induced LH surge.^37^ Groups 2 and 3 received sterile saline (4 µL). At at 8.45 am that same day, the animals in both groups 1 and 2 then received a 3V injection of IGF‐1 as described above, whereas the animals in Group 3 received a 3V injection of sterile saline. On day 4, the injection protocol described for day 3 was repeated, then the animals were killed 30 minutes after (at 9.15 am) their final injection of either IGF‐1 or saline**.** The MBH tissue fragment was dissected from each brain and incubated in Locke's media as detailed above. After 30 minutes of incubation, the MBH tissue weighed a mean ± SEM of 13.8 ± 3.8 mg. The samples were then collected and saved for subsequent assessment of NKB using an ELISA kit purchased from MyBiosource Inc. The total number of animals used was 48.

### 2.7 Enzyme‐linked immunoassays

DYN, NKB, GnRH and B‐ENDO were measured from the media using kits purchased from MyBiosource, Inc. DYN and ß‐ENDO ELISA sensitivities were 1 pg mL^‐1^ and both inter‐ and intra‐assay coefficients of variation percentage (CV%) were <10%. The NKB ELISA sensitivity was 2.0 pg mL^‐1^ and CV% was less than 15%. The GnRH ELISA sensitivity was 0.06 ng mL^‐1^, the inter‐assay CV was ≤8% and the intra‐assay CV% was ≤12%. The sensitivity of the Kp ELISA was 10 pg mL^‐1^ and the intra‐ and inter‐assay CV% was ≤15%.

### 2.8 Western blot analysis

Brain tissues were homogenised in 1% Igepal CA‐630, 20 mmol L^‐1^ Tris‐Cl, pH (8.0), 137 mmol L^‐1^ NaCl, 2 mmol L^‐1^ ethylenediaminetetraacetic acid, 10% glycerol, 10 mmol L^‐1^ sodium pyruvate, 10 mmol L^‐1^ sodium fluoride, 1 mmol L^‐1^ sodium orthovanadate, 1 mmol L^‐1^ phenylmethylsulfonyl fluoride and 0.25% protease inhibitor cocktail (Sigma‐Aldrich) at 4°C. The homogenates were incubated on ice for 30 minutes. After incubation, the homogenates were centrifuged at 12 000 *g* for 15 minutes at 4°C. The protein concentration in the resulting supernatant was measured by the Pierce 660 nm Protein assay kit (Thermo Scientific, Waltham, MA, USA) using bovine serum albumin as standard. The proteins (100 µg) for immunoblotting were electrophoresed through 4%‐20% sodium dodecyl sulphate‐polyacrylamide gel electrophoresis for DYN, KOR‐1, β‐ENDO, NKB, NK3R and Kp under reducing conditions. The separated proteins were electrophoretically transblotted onto polyvinylidene difluoride membranes. Following transfer, membranes were blocked with 5% nonfat dried milk and 1% Tween‐20 in phosphate‐buffered saline (PBS) (pH7.4) for 3 hours and subsequently incubated at 4°C overnight with the appropriate primary antibody: goat anti‐DYN (dilution 1:250; catalogue no.: sc‐46313; RRID:AB_2283705; Santa Cruz Biotechnology, Santa Cruz, CA, USA), mouse anti‐KOR‐1 (dilution 1:250; catalogue no.: sc‐374479; RRID:AB_10989571; Santa Cruz Biotechnology), mouse anti‐β‐ENDO (dilution 1:1000; catalogue no.: ab54205; RRID:AB_879602; Abcam Inc., Cambridge, MA, USA), rabbit anti‐NKB (dilution 1:500; catalogue no.: NB300‐201; RRID:AB_10000783; Novus Biologicals, Centennial, CO, USA), rabbit anti‐NK3R (dilution 1:1000; catalogue no.: NBP1‐00949; RRID:AB_1503775; Novus Biologicals) and rabbit anti‐Kp (3 µg mL^‐1^; catalogue no.: NBP1‐45672; RRID:AB_10009110; Novus Biologicals). After incubation, membranes were washed in PBS/0.1% Tween‐20 and then incubated with horseradish peroxidase‐labelled secondary antibodies (dilution 1:50 000; mouse anti‐goat; catalogue no.: sc‐2354; RRID:AB_628490; goat anti‐mouse; catalogue no.: sc‐2005; RRID:AB_631736; or goat anti‐rabbit secondary antibody; catalogue no.: sc‐2004; RRID:AB_631746; Santa Cruz Biotechnology) for 2 hours at room temperature. Following incubation, membranes were washed in PBS/0.1% Tween‐20. The specific protein signals were visualised by enhanced chemiluminescence (Western Lightning Plus‐ECL; PerkinElmer, Waltham, MA, USA) and quantified with imagej, version 1.43 (http://imagej.nih.gov; RRID:SCR_003070; National Institute of Health, Bethesda, MD, USA). Subsequently, all membranes were also stripped using Re‐Blot Plus kit (EMD Millipore, Burlington, MA, USA) and reprobed with mouse monoclonal antibody to β‐actin (catalogue no.: #A1978; RRID:AB_476692; Sigma‐Aldrich) and goat anti‐mouse (catalogue no.: sc2005; RRID:AB_631736; Santa Cruz Biotechnology) to normalise for the amount of sample loading when appropriate. Following washing, the detection and quantitation of β‐actin was carried out as described above.

### 2.9 Statistical analysis

Data are expressed as the mean ± SEM. An unpaired *t* test was used to detect significant differences between control and the treated groups. A paired *t* test was used when comparing each animal's basal release (media only) to its senktide‐induced release of a specific peptide. Multiple comparisons were performed using ANOVA, with post‐hoc testing using the Student‐Newman‐Keuls multiple range test. Statistical tests were conducted with INSTAT and Prism software (http://www.graph.pad.com; RRID:SCR_002798; GraphPad Software Inc., San Diego, CA, USA). *P* < 0.05 was considered significantly different.

## 3 RESULTS

**Figure 2:**
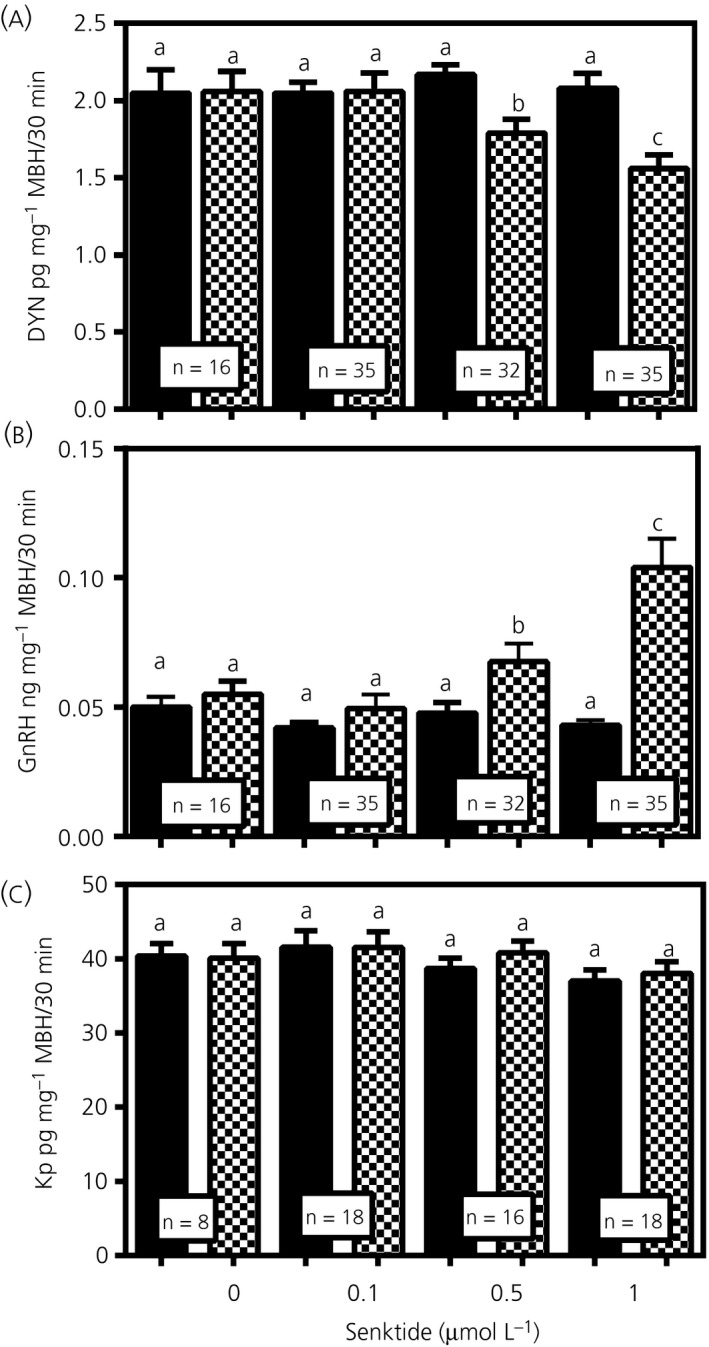
The dose‐response effects of senktide on dynorphin (DYN), gonadotrophin‐releasing hormone (GnRH) and Kp release. A, DYN secretion from medial basal hypothalamus (MBH) tissues incubated in vitro before and after addition of senktide to the medium at doses of 0‐1.0 µmol L^‐1^. These results show that the basal levels of DYN secreted into the media were not different between the incubation groups and that the presence of senktide at doses of 0.5 and 1.0 µmol L^‐1^ caused marked suppressions in DYN secretion. B, These results also demonstrate that the basal levels of GnRH secreted were not different between the incubation groups, although senktide at the doses of 0.5 and 1.0 µmol L^‐1^ caused marked increases in GnRH secretion. C, These results show that Kp secreted into the media was not different between the basal levels or after the addition of senktide. The same media samples were used to assess all of these peptides. Closed bars, basal/medium only; hatched bars, senktide‐treated. The number of animals represented is show within the bars. Bars represent the mean ± SEM. One‐way ANOVA was utilised to compare basal and senktide dose groups and Student Newman Keul’s post‐hoc tests were used to determined *P* values, a vs b, *P* < 0.05; a vs c, *P* < 0.01; b vs c, not significant

Figure 2 demonstrates the release of DYN, GnRH and Kp from MBH tissues incubated in vitro before (basal) and after the addition of senktide to the media. Figure 2A shows that the basal levels (closed bars) of DYN were not significantly (NS) different from each other (NS; *F*_3,114_ = 0.3214), although, once senktide was added, then dose‐responsive differences in DYN secretion were observed. In this regard, media samples without senktide (“0”) or with the 0.1 µmol L^‐1^ dose of senktide were ineffective in altering DYN secretion; however, a suppression of DYN secretion was noted with both the 0.5 µmol L^‐1^ (*P* < 0.05) and 1.0 µmol L^‐1^ (*P* < 0.01; *F*_3,114_ = 8.417) doses of senktide. Figure 2B demonstrates the effects of senktide on GnRH release from the same media samples used for the DYN assessments. Similar to DYN, no differences in basal secretion of the GnRH were observed between any of the groups (NS, *F*_3,114_ = 1.905), and the lowest dose of senktide (0.1 µmol L^‐1^) did not affect the release of GnRH. However, increases in secretion of the GnRH peptide were noted with both the 0.5 µmol L^‐1^ (*P* < 0.01) and the 1.0 µmol L^‐1^ (*P* < 0.001; *F*_3,114_ = 16.68) doses of senktide. Additionally, the remaining media samples were used to assess the effects of senktide on the secretion of Kp and β‐ENDO. Importantly, unlike DYN and GnRH, the addition of the different doses of senktide to the media did not affect the in vitro release of Kp at any of the doses (NS; *F*_3,56_ = 0.8061) (Figure 1C). Likewise, ß‐ENDO (not shown) was also unaffected by the different doses of senktide (NS; *F*_3,56_ = 1.970).

**Figure 3:**
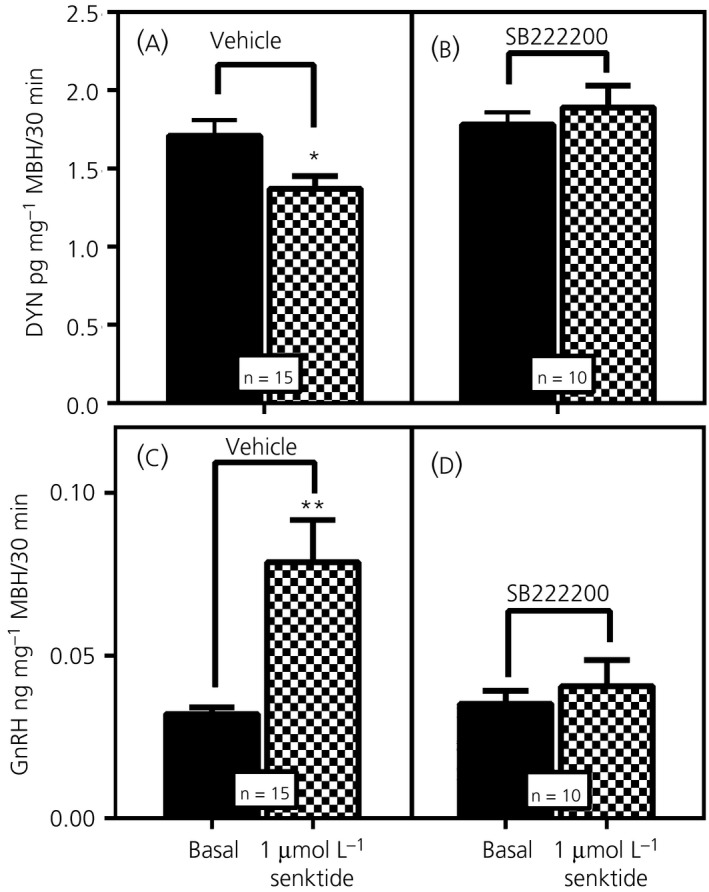
The effect of neurokinin 3 receptor (NK3R) antagonist administered centrally on the subsequent in vitro release of both dynorphin (DYN) and gonadotrophin‐releasing hormone (GnRH). A, DYN secretion in animals that received a third ventricular (3V) injection of vehicle 2 hours prior to the in vitro incubation. After the initial incubation in medium only to establish basal release, senktide was added to the incubation medium, resulting in a suppression in DYN secretion compared to basal. B, DYN secretion in animals that received a 3V injection of the NK3R antagonist, SB222200, 2 hours prior to the in vitro incubation. Note that the basal secretion of DYN was not affected by the prior central administration of SB222200, and was similar to the basal levels of the control animals in (A); however, the antagonist blocked the senktide‐induced decrease of DYN secretion. C, GnRH secretion in the animals that received the 3V injection of vehicle. Senktide induced a marked increase in GnRH release over basal secretion. D, GnRH secretion in the animals that received the 3V injection of the NK3R antagonist. The NK3R antagonist had no effect on basal secretion and that the basal levels were similar to those basal levels shown in (C); however, the antagonist blocked the senktide‐induced release of GnRH. The same media samples were used to assess both peptides. The number of animals represented is shown within the bars. Bars represent the mean ± SEM. Closed bars, basal/medium only; hatched bars, senktide‐treated. Paired *t* test comparing basal/medium only vs senktide‐induced medium from the same animal tissues was used to determine *P* values: *P < 0.05; **P < 0.01. MBH, medial basal hypothalamus

A specific study was conducted to assess whether an NK3R antagonist would block the senktide‐induced actions on DYN and GnRH secretions from the MBH. Figure 3A shows DYN secretion in animals that received a 3V injection of vehicle 2 hours prior to the time MBH tissues were collected for subsequent in vitro incubation. After an initial incubation period to establish basal release, the addition of senktide to the incubation medium caused a suppression (*P* < 0.05, *t* = 2.71, *df* = 12) in DYN secretion compared to basal levels. Figure 3B shows DYN secretion in animals that received a 3V injection of the NK3R antagonist (SB222200) 2 hours prior to the in vitro incubation. Note that the basal levels of the SB222200‐treated animals (Figure 3B) are similar to basal levels of the vehicle‐treated animals shown in Figure 3A. The senktide‐induced decrease in DYN secretion (seen in Figure 3A) was blocked by the SB22002 administration (*P* = 0.278, *t* = 1.17, *df* = 9) shown in Figure 3B. GnRH release was measured from these same samples and shows that the basal secretion from the SB222200‐treated animals was similar to the vehicle‐treated animals. Figure 3C shows that senktide induced an increase in GnRH release (*P* < 0.01, *t* = 2.94, *df* = 12), whereas Figure 3D demonstrates that this action to stimulate GnRH release was blocked (*P* = 0.243, *t* = 1.25, *df* = 9) by the NK3R antagonist.

**Figure 4:**
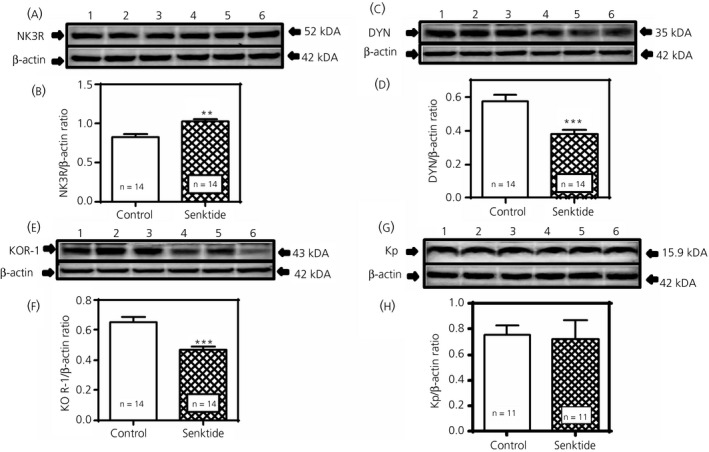
Effects of senktide administered centrally for 4 days on neurokinin 3 receptor (NK3R), dynorphin (DYN), kappa opioid receptor (KOR‐1) and Kp protein expression in the medial basal hypothalamus (MBH). A, Representative western blot of NK3R and β‐actin proteins from animals that received either a daily third ventricular (3V) injection of saline (lanes 1‐3) or senktide (lanes 4‐6). B, Each bar represents the mean ± SEM of the densitometric quantification of all bands assessing NK3R protein normalised to β‐actin protein. C, Representative western blot of DYN and ß‐actin proteins from animals that received either saline (lanes 1‐3) or senktide (lanes 4‐6). D, Each bar represents the mean ± SEM of the densitometric quantification of all bands assessing DYN protein normalised to ß‐actin protein. E, Representative western blot of KOR‐1 and ß‐actin proteins from animals that received either saline (lanes 1‐3) or senktide (lanes 4‐6). F, Each bar represents the mean ± SEM of the densitometric quantification of all bands assessing KOR‐1 protein normalised to ß‐actin protein. G, Representative western blot of Kp and β‐actin proteins from animals that received either of saline (lanes 1‐3) or senktide (lanes 4‐6). H, Each bar represents the mean ± SEM of the densitometric quantification of all bands assessing Kp protein normalised to β‐actin protein. Note that senktide induced an increase in NK3R protein expression, although it suppressed DYN and KOR‐1 protein expression over the levels of their respective saline controls. The number of animals represented is shown within each bar. Open bars, saline controls; hatched bars, senktide‐treated. An unpaired *t* test was used to compare control vs senktide‐treated animal groups: **P < 0.01, ***P < 0.001

Figure 4 demonstrates the effects of central administration of senktide for 4 days on NK3R, DYN, KOR‐1 and Kp protein expression in the MBH. Specifically, Figure 4A,B show that senktide delivered into the 3V induced an increase (*P* < 0.01, *t* = 3.14, *df* = 24) in NK3R protein expression over the levels shown for the control animals that received 3V injections of saline. Figure 4C,D depicts a senktide‐induced suppression (*P* < 0.001; *t* = 4.096, *df* = 26) in DYN protein compared to control levels and, furthermore, Figure 4E,F demonstrates the suppressed (*P* < 0.001; *t* = 4.380, *df* = 24) expression of KOR‐1 in the senktide‐treated animals. Additionally, Figure 4G,H demonstrates that Kp synthesis was not affected (*t* = 0.2794, *df* = 20) by senktide administration. Furthermore, ß‐ENDO protein expression (not shown) was unaffected by the senktide (Control: 0.98 ± 0.03 vs Senktide: 0.90 ± 0.05; N = 7, *P* = 0.222, *t* = 1.297, *df* = 12).

**Figure 5:**
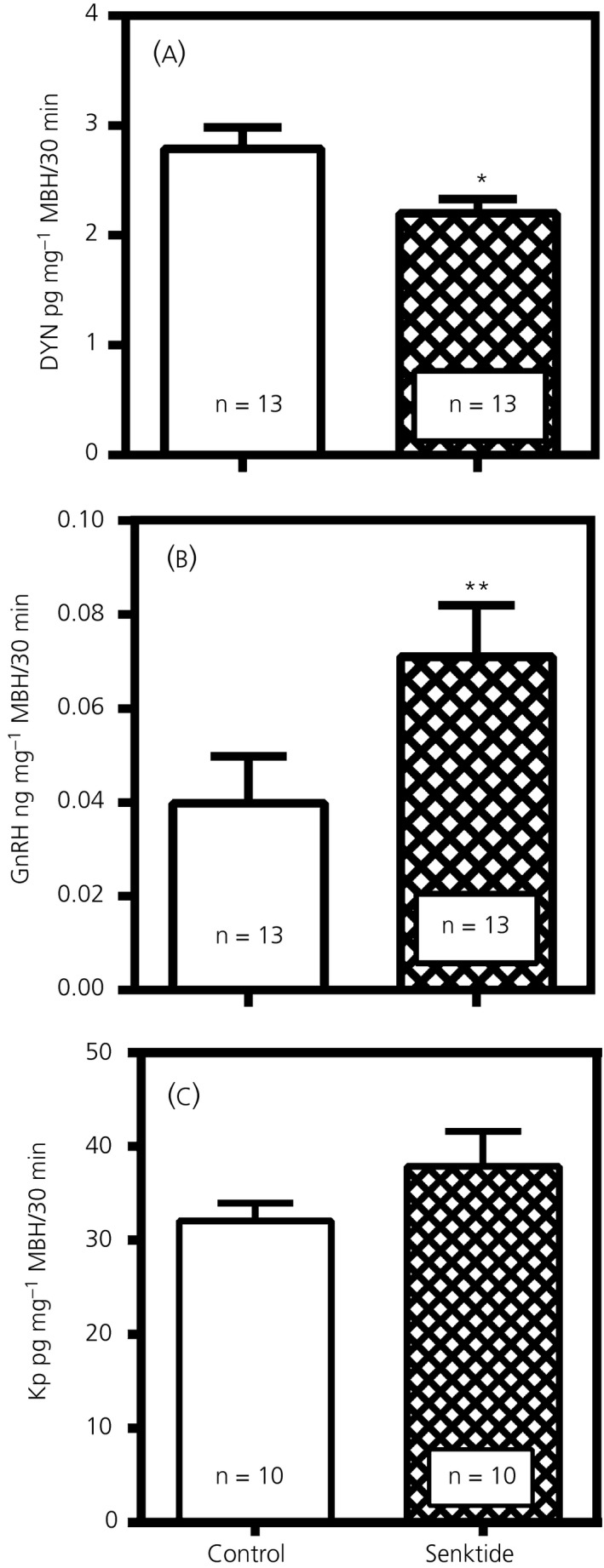
Effects of senktide administered centrally for 4 days on the subsequent secretion of dynorphin (DYN), gonadotrophin‐releasing hormone (GnRH) and Kp from medial basal hypothalamus (MBH) tissues incubated in vitro. A, The suppressed release of DYN in the animals that received a daily third ventricular (3V) injection of senktide compared to the saline‐injected control animals. B, The elevated secretion of GnRH in the senktide‐treated animals compared to the saline‐injected control animals. C, Senktide administration was ineffective at inducing Kp secretion in the senktide‐treated animals compared to the saline‐injected control animals. The number of animals represented is shown within each bar. Bars represent the mean ± SEM. Open bars, saline controls; hatched bars, senktide‐treated. An unpaired *t* test was used to compare control vs senktide‐treated animal groups: *P < 0.05, **P < 0.01

Figure 5 illustrates the effect of senktide on DYN, GnRH and Kp secretion when MBH tissues were incubated in vitro following 4 days of senktide delivery into the 3V. Figure 5A demonstrates that compared to the saline‐injected control animals, the animals injected with senktide revealed suppressed (*P* < 0.05, *t* = 2.284, *df* = 24) DYN release. Conversely, Figure 5B clearly shows elevated (*P* < 0.01, *t* = 2.844, *df* = 24) GnRH release compared to the control levels. In the remaining media samples, we observed that Kp release was not affected by senktide administration (*t* = 1.418, *df* = 18) (Figure 5C). Likewise, no differences in peptide secretion were noted for β‐ENDO (Control: 4.60 ± 0.52, n = 12 vs Senktide: 4.85 ± 0.59; *t* = 0.313, *df* = 24, n = 14).

**Figure 6:**
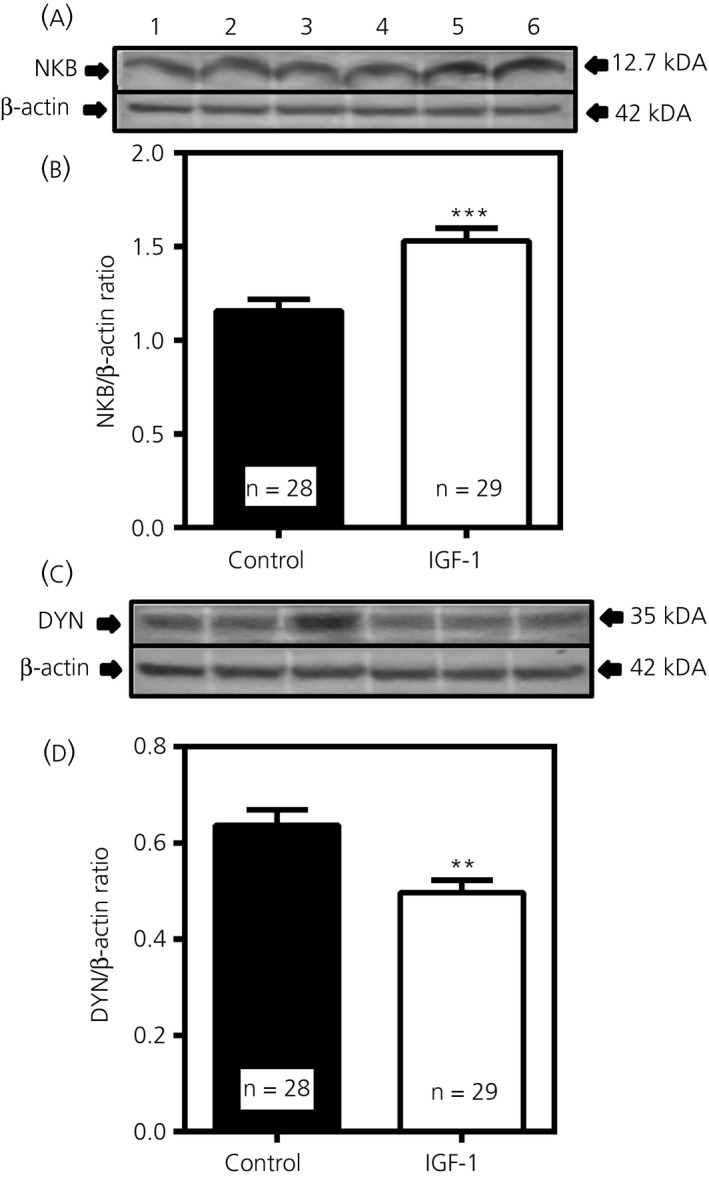
Effects of insulin‐like growth factor (IGF)‐1 administered centrally for 4 days on neurokinin B (NKB) and dynorphin (DYN) protein expression in the medial basal hypothalamus. A, Representative western blot of NKB and β‐actin proteins from animals that received either a daily third ventricular (3V) injection of saline (lanes 1‐3) or IGF‐1 (lanes 4‐6). B, Each bar represents the mean ± SEM of the densitometric quantification of all bands assessing NKB protein normalised to β‐actin protein. C, Representative western blot of DYN and ß‐actin proteins from animals that received either saline (lanes 1‐3) or IGF‐1 (lanes 4‐6). D, Densitometric quantification of all bands assessing DYN protein normalised to ß‐actin protein. Note that IGF‐1 induced an increase in NKB protein expression, although it suppressed DYN protein expression over the levels of their respective saline controls. The number of animals represented is shown within each bar. Closed bars, saline controls; open bars, IGF‐1‐treated. An unpaired *t* test was used to compare control vs senktide‐treated animal groups: **P < 0.01, ***P < 0.001

The central administration of IGF‐1 for 4 days revealed altered NKB and DYN protein expression in the MBH. In this regard, Figure 6A,B demonstrates that IGF‐1 delivered into the 3V induced an increase (*P* < 0.001; *t* = 4.125, *df* = 56) in NKB protein expression over levels depicted for the control animals that received 3V injections of saline. Figure 6C,D shows the level of DYN protein expression was suppressed (*P* < 0.01; *t* = 3.358, *df* = 54) in the animals that received the IGF‐1. No differences were detected in peptide expression for either ß‐ENDO (Control: 0.58 ± 0.05, n = 8 vs IGF‐1: 0.63 ± 0.04; n = 8; *t* = 0.8359, *df* = 14) or Kp (Control: 1.39 ± 0.05, n = 7 vs IGF‐1:1.35 ± 0.04; n = 7, *t* = 0.6644, *df* = 12).

**Figure 7:**
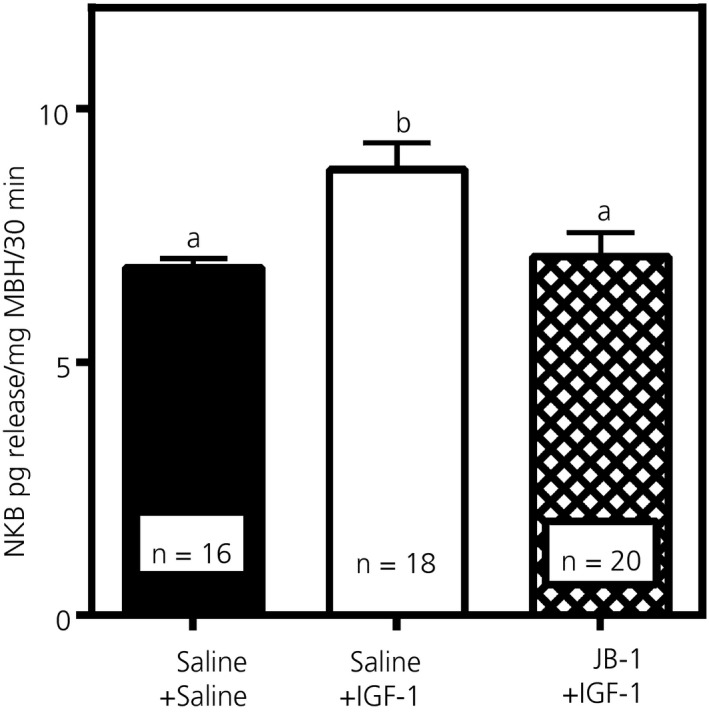
Effects of insulin‐like growth factor (IGF)‐1 administered centrally for 4 days on the subsequent secretion of neurokinin B (NKB) from medial basal hypothalamus (MBH) tissues incubated in vitro. Animals received either a daily third ventricular (3V) injection of saline or IGF‐1. Additionally, a third group of animals received IGF‐1 plus a 3V injection of JB‐1, the IGF‐1R antagonist, during the last 2 days of the injection schedule. For more details on injection and in vitro incubation procedures, see Figure 1. Note that the JB‐1 blocked the IGF‐1 induced release of NKB. The number of animals represented is shown within each bar. Bars represent the mean ± SEM. Closed bar, saline control, n = 16; open bar, IGF‐1‐treated, n = 18; hatched bar, IGF‐1 + JB‐1, n = 20. One‐way ANOVA with Student‐Neuman Keuls post‐hoc test determined the *P* values, a vs b, *P* < 0.01

Figure 7 shows that the release of NKB was enhanced (*P* < 0.01, *F*_2,53_ = 5.930) from MBH tissues that were incubated in vitro following 4 days of IGF‐1 delivery into the 3V and that this stimulatory action of IGF‐1 was blocked by the IGF‐1R antagonist, JB‐1. In these same media samples, we also observed that IGF‐1 did not stimulate secretion of Kp (Control: 37.2 ± 2.1; n = 16 vs IGF‐1: 39.7 ± 1.3; n = 20, *t* = 1.221; *df* = 34).

## 4 DISCUSSION

Because DYN is a known inhibitor of GnRH secretion,^4^, ^5^ we assessed, for the first time, the upstream control of DYN synthesis and release within the prepubertal MBH. In this brain region, DYN, NKB and Kp are produced by neurones localised within the ARC. Although co‐localisation of these peptides exists in subpopulations of these neurones, the extent of their overlap varies between species.^4^, ^5^, ^20^, ^21^, ^38^ We initially assessed the interactions between NKB and DYN because most DYN neurones in the MBH express the NK3R^38^ **;** hence, suggesting that NKB likely contributes to the regulation of prepubertal DYN secretion. In the present study, we considered the possibility that NKB, which increases in the MBH as puberty approaches,^22^ inhibits DYN release and that this action, at least in part, would suppress the DYN inhibitory tone on GnRH and facilitate release of the GnRH peptide at the onset of puberty. The results of the present study support this; however, because there are several regions of the hypothalamus that co‐express DYN and NK3R,^38^ the study design did not identify which specific populations of these neurones were affected. In this regard, the acute exposure of prepubertal MBH tissue explants to senktide in vitro caused a dose‐dependent inhibition in DYN release that coincided with an increase in GnRH secretion. Importantly, both responses were blocked by the prior central administration of an NKB receptor antagonist known to delay the onset of puberty in the female rat^32^ and block the NKB stimulation of LH release**.** 36 Long‐term effects of senktide were assessed following 4 days of administration into the third ventricle of late juvenile female rats. In this regard, we observed an increase in protein expression of the NK3R within the MBH, along with suppressed protein expression of DYN and its receptor, KOR‐1. Furthermore, in vitro incubation of MBH tissues following the 4 days of senktide administration in vivo resulted in suppressed DYN and increased GnRH secretion. It is important to note that senktide did not affect β‐ENDO**,** another endogenous opioid inhibitor of GnRH,^7^ in any of our assessments. Taken together, our results suggest that activation of the NK3R can cause a suppression of the DYN brake on prepubertal GnRH secretion in female rats and, at least in part, allow for the rise in the GnRH peptide to occur. Because NK3R are localised on GnRH nerve fibres in the MBH^39^, ^40^, ^41^ it is possible, if not likely, that GnRH was also released by a direct action of senktide on these nerve fibres. Earlier studies in rats have produced varied results showing that NKB can either stimulate or inhibit GnRH/LH secretion, often depending on species, sex and age. The present study clearly shows a stimulatory action of senktide/NKB to release GnRH which supports previous studies showing increased GnRH/LH release in prepubertal female rats^23^ sheep^42^ and rhesus monkeys,^24^ as well as in adult sheep^43^ and mice.^41^

Because Kp is also synthesised by the neurones in the ARC,^44^, ^45^ we assessed the potential for NKB to regulate this puberty‐related peptide as well. In this regard, we did not observe any senktide‐induced changes in Kp synthesis or release from the same late juvenile female rats that were used for the above mentioned DYN/GnRH assessments, hence indicating that Kp was not involved in the senktide‐induced release of GnRH. A previous report, however, demonstrated that senktide infused into the stalk median eminence stimulated Kp release in prepubertal female monkeys, although the release was markedly less than that produced in pubertal monkeys,^24^ an effect considered to be a result of differences in circulating oestradiol levels. Therefore, the inability of senktide to stimulate Kp release in the present study could be the result of a species difference or to the low levels of oestradiol in late juvenile female rats. Interestingly, this previous study also showed that NKB and Kp take independent signalling paths to affect the release of GnRH in prepubertal female monkeys.^24^ Another report using tissue slices from adult male transgenic mice also indicated that NK3R activated GnRH release was Kp‐independent.^41^ Thus, the present study, conducted in immature female rats and showing that senktide/NKB administration resulted in increased GnRH without affecting Kp release, also supports the notion that NK3R activated GnRH release is independent of Kp, at least at this phase of development.

After determining pubertal changes in DYN and GnRH generated by prepubertal senktide/NKB stimulation, we considered the possibility that a peripheral, metabolic signal could be an upstream regulator of endogenous NKB synthesis and release. Several studies collectively have suggested that IGF‐1 would be a likely candidate for such a role. The circulating levels of this peptide rise strikingly during puberty in rodents^26^, ^46^ ruminants,^47^, ^48^ monkeys^49^ and humans**.** 50 The peptide is capable of crossing the blood‐brain barrier and binds to high levels of the IGF‐1R within the MBH.^51^, ^52^, ^53^ In late prepubertal female rats, an increase in hepatic synthesis of IGF‐1 corresponded to an increase in serum IGF‐1, whereas the central synthesis of the peptide did not change.^26^ Importantly, over the years, we have clearly shown that IGF‐1 is capable of stimulating GnRH and LH release in female rats^25^, ^26^ during prepubertal and peripubertal development through a centrally‐mediated action. Additionally, repeated exposure of the hypothalamus to increased levels of IGF‐1 effectively accelerates the timing of puberty in rodents and primates,^26^, ^27^, ^28^ whereas, in both species, alterations in the synthesis of IGF‐1 by exposure to endocrine disrupters can delay puberty.^15^, ^36^, ^54^ Also, IGF‐1 replacement can restore puberty in GH‐deficient mice^55^ and in rats exposed to lead.^56^ Although these studies depict a direct role for IGF‐1 in the pubertal release of GnRH, it is also possible that there are other mechanisms by which this peptide can facilitate GnRH secretion at the time of puberty. The present study supports this hypothesis by showing an early action of IGF‐1 with respect to regulating NKB. Specifically, the repeated central exposure to IGF‐1 for 4 days caused increased NKB and decreased DYN protein expression within the MBH without affecting the expression of Kp. This absence of an IGF‐1 effect on Kp synthesis within the MBH supports a previous study.^29^ Furthermore, after the 4 days of IGF‐1 administration, there was no change in the release of Kp in vitro, although there was an increase in the release of NKB, an action that was blocked by the IGF‐1R antagonist, JB‐1. The exact site of this IGF‐1‐NKB interaction has yet to be determined because it is not known whether NKB neurones express IGF‐1Rs or whether the IGF‐1 is binding to IGF‐1Rs expressed on an interneurone.

The results described above depict early critical responses to IGF‐1 within the MBH that are associated with the onset of female puberty in the rat. Although the rostral hypothalamic area (RHA), which includes the anteroventral periventricular nucleus, is indeed important, we suggest that, at least in the rat animal model, the initial or early actions related to the onset of puberty occur in the MBH. In this regard, IGF‐1 can not only stimulate prepubertal GnRH release directly from the nerve terminals in the median eminence,^25^ but, as we have described above, it can also stimulate secretion of NKB. Once released then NKB is capable of a dual action. First, it binds to the NK3R on GnRH nerve terminals^40^, ^41^ and can stimulate prepubertal GnRH/LH secretion.^23^, ^24^ Second, after also binding to NK3R on DYNergic neurones in the ARC, it then inhibits DYN secretion and therefore contributes to the removal of the DYN inhibition of prepubertal GnRH release, thus allowing for further release of the peptide. We suggest that the increase in GnRH release at the onset of puberty is due, at least in part, to these early prepubertal responses to IGF‐1 within the MBH during late juvenile development. Interestingly, the present study also revealed that these early effects were not associated with any changes in the synthesis or secretion of Kp within the MBH. As pubertal development proceeds, however, the Kp synthesising neurones in the RHA begin responding to the rising levels of oestradiol 22, 57, 58 and IGF‐1.^25^, ^26^, ^59^ In support of this, we have shown that, although IGF‐1 was not capable of inducing Kp release from RHA explants obtained during late juvenile development, when oestradiol levels are low, the peptide was able to stimulate release of Kp from the explants removed from animals in their first pro‐oestrus.^60^ Importantly, Kp is known to be a potent stimulator of GnRH release,^61^, ^62^, ^63^ especially during pro‐oestrus,^64^, ^65^, ^66^ when oestrogen levels are increasing before the GnRH/LH surge. It is critical to note that the Kp producing neurones within the RHA not only send some of their nerve processes caudally to the MBH, but also project processes to adjacent GnRH synthesising neurones in other areas of the RHA and in the preoptic area^40^, ^67^, ^68^ that express Kp receptors. Evidence indicates that, once Kp is released within the RHA,^59^ it acts directly on these GnRH neurones via its receptor^69^, ^70^, ^71^ to stimulate the synthesis and secretion of the GnRH peptide and thus, drive the pubertal process**.** Collectively, the results of these studies assessing both the MBH and the RHA indicate that the earliest actions to initiate GnRH release at the onset of puberty occur within MBH.

**Figure 8:**
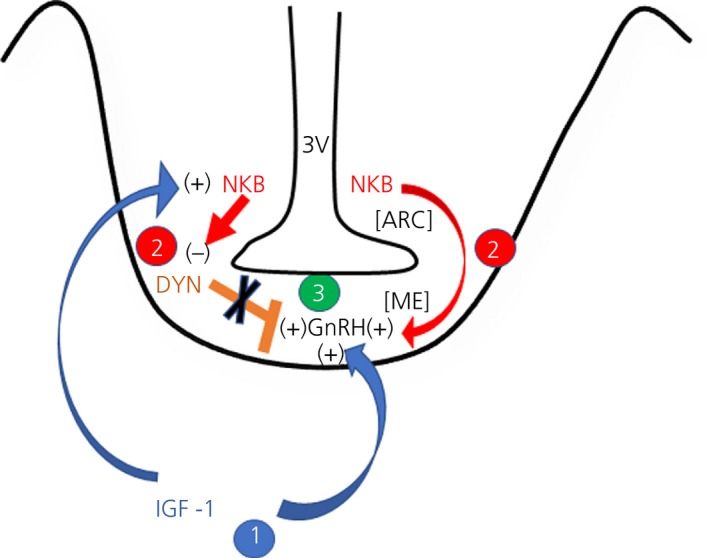
Schematic showing insulin‐like growth factor (IGF)‐1, neurokinin B (NKB) and dynorphin (DYN) actions contributing to the prepubertal regulation of gonadotrophin‐releasing hormone (GnRH) secretion. 1. Blue arrows indicate that IGF‐1 directly stimulates GnRH release from the median eminence (ME), at the same time as stimulating NKB synthesis and release from neurones in the arcuate nucleus (ARC). 2. Red arrows indicate that NKB likewise stimulates GnRH release from the ME, at the same time as inhibiting DYN (orange bar) synthesis and release from neurones in the ARC. 3. The combined actions of IGF‐1, NKB and DYN result in increased GnRH secretion during the initiation of puberty. 3V, third ventricle; +, stimulation; −, inhibition

In summary, the present study demonstrates, for the first time, an IGF‐1/NKB/DYN pathway regulating GnRH secretion at the time of puberty. Specifically, we have shown that the repeated central administration of IGF‐1 induces prepubertal NKB synthesis and secretion within the MBH. Also, we have revealed that the central administration of senktide, an NKB agonist, resulted in the suppressed synthesis of DYN and its KOR‐1 receptor**.** Furthermore, senktide inhibited DYN secretion, an action associated with the increased secretion of GnRH. Additionally, none of these early actions were associated with changes in Kp synthesis or release. These results indicate that this newly described pathway contributes to the removal of the DYN inhibitory tone on prepubertal GnRH secretion and thus allows for the puberty‐related increase in the release of the peptide to begin. Furthermore, we suggest that this action, together with the previously stated ability of both NKB and IGF‐1 to independently stimulate GnRH release directly from the nerve terminals, facilitates the initial increased release of GnRH at the onset of puberty. Figure 8 depicts a schematic showing the actions and interactions of IGF‐1, NKB and DYN on prepubertal GnRH release.

## CONFLICT OF INTERESTS

The authors declare that they have no conflicts of interest.

## Data Availability

The data that support the findings of this study are openly available in Pubmed Central at http://ncbi.nlm.nih.gov/pmc according to the National Institutes of Health public access policy.
